# Spatiotemporal Expression of SphK1 and S1PR2 in the Hippocampus of Pilocarpine Rat Model and the Epileptic Foci of Temporal Lobe Epilepsy

**DOI:** 10.3389/fcell.2020.00800

**Published:** 2020-10-08

**Authors:** Yuan-Yuan Dong, Min Xia, Lin Wang, Shuai Cui, Qiu-Bo Li, Jun-Chen Zhang, Shu-Shu Meng, Yan-Ke Zhang, Qing-Xia Kong

**Affiliations:** ^1^Department of Neurology, Affiliated Hospital of Jining Medical University, Jining, China; ^2^Department of Surgery, Weifang Medical University, Weifang, China; ^3^Department of Pediatrics, Affiliated Hospital of Jining Medical University, Jining, China; ^4^Department of Neurosurgery, Affiliated Hospital of Jining Medical University, Jining, China; ^5^Qingdao West Coast New Area Central Hospital, Qingdao, China

**Keywords:** temporal lobe epilepsy, SphK1, S1PR2, astrocyte, neuron

## Abstract

Temporal lobe epilepsy (TLE) is a severe chronic neurological disease caused by abnormal discharge of neurons in the brain and seriously affect the long-term life quality of patients. Currently, new insights into the pathogenesis of TLE are urgently needed to provide more personalized and effective therapeutic strategies. Accumulating evidence suggests that sphingosine kinase 1 (SphK1)/sphingosine 1-phosphate receptor 2 (S1PR2) signaling pathway plays a pivotal role in central nervous system (CNS) diseases. However, the precise altered expression of SphK1 and S1PR2 in TLE is remaining obscure. Here, we have confirmed the expression of SphK1 and S1PR2 in the pilocarpine-induced epileptic rat hippocampus and report for the first time the expression of SphK1 and S1PR2 in the temporal cortex of TLE patients. We found an increased expression of SphK1 in the brain from both epileptic rats and TLE patients. Conversely, S1PR2 expression level was markedly decreased. We further investigated the localization of SphK1 and S1PR2 in epileptic brains. Our study showed that both SphK1 and S1PR2 co-localized with activated astrocytes and neurons. Surprisingly, we observed different subcellular localization of SphK1 and S1PR2 in epileptic brain specimens. Taken together, our study suggests that the alteration of the SphK1/S1PR2 signaling axis is closely associated with the course of TLE and provides a new target for the treatment of TLE.

## Introduction

Epilepsy is a common neurological disease characterized by recurrent spontaneous epileptic seizures. According to recent data, epilepsy affects about 65 million people worldwide (Moshé et al., 2015). Temporal lobe epilepsy (TLE) is the most common pharmacoresistant epilepsy type, which accounts for 30–40% of newly diagnosed epilepsy patients (Kwan and Sander, 2004). Numerous neuropathological findings have shown that hippocampal sclerosis (HS) is a representative histopathological alteration in patients with refractory epilepsy, including neuronal loss and gliosis (Lanerolle et al., 2010). Astrocyte, the major glial cell type of mammalian central nervous system (CNS), whose activation is an important cause of chronic epilepsy (Hong et al., 2019). Gliosis, aberrant neurogenesis, and hippocampal network plasticity contributed to an epileptic focus and was considered to involve in the epileptogenesis of TLE (Mathern et al., 1996; Buckmaster and Dudek, 1997; Parent et al., 1997; Robel et al., 2015).

Sphingosine kinase 1 (SphK1) is a key enzyme that catalyzes sphingosine into sphingosine-1-phosphate (S1P), which is located in hippocampal neurons, astrocytes, microglia, and cerebellar granule cells in CNS (Anelli et al., 2005; Yang et al., 2018). S1P acts as a bioactive molecule in an autocrine or paracrine manner that is mediated by five high-affinity G protein-coupled receptors (S1PR1–S1PR5) on the cell membrane, thus regulating a complex network of intracellular signaling pathways (Herr and Chun, 2007; Chun et al., 2010). Recent research on the role of SphK1/S1P receptor signaling has shown that this plays a pivotal role in onset and progression of CNS disorders; for example, the suppression of SphK1 can decrease the production of IL-17 and relieve neuronal damage induced by microglia in cerebral ischemia-reperfusion (IR) or an *in vitro* oxygen-glucose deprivation reperfusion system, and S1P levels affect hippocampal neuronal cell fate after transient global cerebral ischemia (tGCI) (Su et al., 2017; Rashad et al., 2018). Furthermore, FTY-720, a sphingosine analog that inhibits SphK1 (Paugh et al., 2003), has been used clinically for the treatment of multiple sclerosis as an immune modulatory drug (Racine, 1972). Recently, S1P receptor subtype 2 (S1PR2) activity has been reported to increase the risk of autoimmunity and epileptogenesis (Akahoshi et al., 2011). SphK1 was a critical signal molecular upstream of S1PR2 in the SphK1/S1P receptor signaling pathway in the modulation of proliferation, survival, and apoptosis in many cell types. We have previously described partial expression characteristics of SphK1 and S1PR2 in a pilocarpine rat model, especially in hippocampal astrocytes (Dong et al., 2019). However, the distribution of SphK1 and S1PR2 in pilocarpine epileptic rats and TLE patients have not yet been systematically elucidated.

The study of epilepsy has been dependent on the use of animal models and brain tissues from the patients. Pilocarpine model replicates several phenomenological features of human TLE and is widely used to study the underlying mechanisms of epileptogenesis (Fisher, 1989; Cui et al., 2019). In the present study, we aimed to determine the alteration of SphK1 and S1PR2 expression in the hippocampus of pilocarpine epileptic rats and the brain tissue of TLE patients and focus on the preferential localization of SphK1 and S1PR2 in astrocytes and neurons.

## Materials and Methods

### Experimental Animals

Adult male Sprague-Dawley (SD) rats (8 weeks, 220–240 g, Shandong Lukang Pharmaceutical Co., Ltd.) were used for animal experiments. Rats were randomly divided into control and experimental groups. The Jining Medical University Animal Ethics Commission approved animal studies and all procedures conformed to the National Institutes of Health Guide for the Care and Use of Laboratory Animals (NIH Publications No. 8023, revised 1978). Rats were fed with standard water/chow and housed in a constant temperature (24 ± 1°C) house and relative humidity (55 ± 5%), under a fixed 12-h light/dark cycle. Rats were euthanized by intramuscular injection of ketamine (10 mg/kg).

### Electrophysiology

Rats were anesthetized by an injection of 10% chloral hydrate (300 mg/kg, i.p.) and stabilized in a stereotactic apparatus (RWD Life Science, Co., Ltd., Shenzhen, China). Two stainless steel screws were implanted as reference electrodes into focal lobe skulls using dental cement. The recording electrodes were implanted into the right dorsal hippocampus (2.8 mm lateral and 3.6 mm posterior to bregma) with a depth of 3.6 mm underneath the dura mater (Budantsev et al., 1993; Zhang et al., 2017). The local field potentials (LFPs) were recorded using a multichannel acquisition processor system (Plexon, Dallas, TX, United States). Signals were filtered between 0.1 and 1000 Hz, amplified (1000×) and digitized at 4 kHz, as previously described (Xu et al., 2016). LFPs signals were analyzed with the NeuroExplorer^®^ v4.0 system. The following experiments were performed 7 days after electrodes implantation.

### Lithium-Pilocarpine Rat Model

A robust convulsive epileptic seizure was induced in rats by intraperitoneal injection (i.p.) of pilocarpine hydrochloride (30 mg/kg; Sigma, United States), 20 h following the injection of lithium chloride (LiCl) (127 mg/kg, i.p.; Sigma, United States). Scopolamine methyl bromide (1 mg/kg, i.p.; Sigma, United States) was administered 30 min before pilocarpine administration to prevent peripheral cholinergic effects. Seizure severity was evaluated by using the Racine Scale. The control rats were injected with respective of 0.9% saline (Racine, 1972). Electrographic seizure activity was defined as the appearance of high amplitude (>2 times baseline), fast activity (>5 Hz) that lasted more than 5 s. In rats with status epilepticus (SE), 0.5% diazepam (10 mg/kg) was administered to terminate their seizures 60 min after the onset of the motor seizure. Control rats and rats that reached stage IV and V after the epileptogenic insults were sacrificed by 10% chloral hydrate overdose at different time points.

### Patient Tissue Collection and Clinical Characteristics

The diagnosis of drug-refractory TLE was based on the International League Against Epilepsy (ILAE) criteria (Engel and International League Against Epilepsy, 2001). The brain samples from sixteen drug-resistant TLE patients and ten control samples (from 2016 to 2019) in this study, primarily from the temporal cortex, were collected from the Neurological Department lab’s established brain tissue bank at the Affiliated Hospital of Jining Medical University. Our study received prior approval by the Ethics Committee of Affiliated Hospital of Jining Medical University, and all patients signed an informed consent. All experiments adhered strictly to the declaration of Helsinki.

All TLE patients underwent extensive presurgical evaluation including a detailed history, a complete general and neurological physical examination, long term video electroencephalogram (EEG) monitoring, neuropsychological testing, and anatomical evaluation by Magnetic Resonance Imaging (3.0T, Siemens, Germany) and PET-CT (GE, United States). These TLE patients experienced three or more seizures per month. Control study used histologically normal temporal cortex from ten patients treated for severe intracranial hypertension after traumatic brain injury (Zhang et al., 2017; Lu et al., 2019). These patients had no seizures after trauma, no history of epilepsy, no exposure to anti-epilepsy drugs or other neurological diseases. The clinical details are summarized in Tables 1, 2.

**TABLE 1:** Clinical features of patients with drug resistance TLE.

Patient	Gender	Age (year)	Duration (year)	AEDs	Localization
El	F	27	11	TPM, VPA, CBZ	LTN
E2	M	29	20	OXC, VPA, LTG	LTN
E3	F	33	10	VPA,CBZ,PHT	RTN
E4	M	17	9	VPA, CBZ, TPM	RTN
E5	M	17	10	VPA, LTG, CBZ	RTN
E6	F	33	3	TPM, OXC	RTN
E7	M	8	7	VPA, CBZ, PB	RTN
E8	F	14	3	VPA, CBZ, TPM	LTN
E9	M	55	5	CZP, PB, VPA	RTN
E10	F	39	7	LTG, CBZ, VPA	RTN
Ell	M	37	20	VPA TPM, OXC	LTN
E12	M	34	20	VPA, OXC	RTN
E13	F	16	13	OXC, LEV, TPM	LTN
E14	M	24	5	CBZ, PHT, PB	RTN
E15	F	36	30	VPA, OXC	RTN
E16	M	7	6	VPA, PHT, CBZ	RTN

*M, male; F, female; AEDs, antiepileptic drugs; TPM, topiramate; VPA, valproic acid; CBZ, carbamazepine; OXC, oxcarbazepine; LTG, lamotrigine; PHT, phenytoin; PB, phenobarbital; LEV, levetiracetam; LTN, left temporal cortex; RTN, right temporal cortex.*

**TABLE 2:** Clinical features of the non-epileptic controls.

Patients	Gender	Age (years)	History of AEDs	Localization	Pathology
Cl	M	27	None	LTN	Normal
C2	F	18	None	RTN	Normal
C3	M	34	None	RTN	Normal
C4	F	30	None	LTN	Normal
C5	F	16	None	RTN	Normal
C6	M	23	None	RTN	Normal
C7	F	37	None	LTN	Normal
C8	M	43	None	RTN	Normal
C9	M	22	None	RTN	Normal
C10	M	20	None	LTN	Normal

*M, male; F, female; LTN, left temporal cortex; RTN, right temporal cortex.*

### Brain Tissue Preparation

Rats used for histochemical staining were deeply anesthetized by intraperitoneal injection of chloral hydrate (300 mg/kg) and transcardially perfused with heparinized 0.9% NaCl solution followed by cold 4% paraformaldehyde (PFA) in 0.1 M PBS. Brains were extracted and postfixed in 4% PFA overnight and then transferred to 30% sucrose in 0.1 M PBS at 4°C until they sank. The coronal brain sections were prepared at the level of the hippocampus. One 5 μm slice was taken every 25 μm. Rats prepared for Western blot analysis were anesthetized with 10% chloral hydrate (300 mg/kg, i.p.), and the brain was then extracted after sacrificed. Fresh hippocampal tissues was dissected from the brain, rapidly frozen in liquid nitrogen, and stored at −80°C until use. The hippocampal sample of each rat was homogenized in pre-cooled RIPA lysis buffer containing 1 mM phenylmethane sulphonyl fluoride (PMSF). The tissue mixtures were vortexed incubated on ice for 10 min. The supernatants were aliquoted after centrifugation at 12000 rpm for 20 min at 4°C and stored at −80°C for subsequent Western blotting. Before Western blotting, total protein concentrations were qualified by the BCA Protein Assay Kit (Beyotime Biotechnology, China).

Human brain tissues were collected immediately after resection and fixed overnight in 4% buffered paraformaldehyde, cryoprotected in 30% sucrose in 0.1M phosphate buffer (PH = 7.40) for 48 h, and stored at −20°C. The fixed human brain tissue was then sectioned at a slice thickness of 5 μm for immunohistochemical staining and double-immunofluorescence labeling. Tissue section slides were stored at −80°C until use.

### Western Blotting Analysis

SDS-PAGE sample loading buffer (5X) was added to the protein samples at a volume ratio of 4:1 before incubating them at 98°C for 5 min. Equal amounts of protein extracts (30 μg) were loaded into each lane of 5–12% gradient precast acrylamide SDS-PAGE gel and were separated at 80–110 V for 2 h. After electrophoresis, separated proteins were electro-transferred onto 0.45 μm polyvinylidene difluoride (PVDF) membranes using a constant voltage of 100 V for 1 h. Membranes were blocked in 5% non-fat milk in Tris-buffered saline (TBS) containing 0.1% Tween 20 for 1 h at room temperature and then incubated with monoclonal mouse anti-SphK1 antibody (Santa Cruz Biotechnology, CA, United States; used at 1:1000), mouse anti-S1PR2 antibody (Santa Cruz Biotechnology, CA, United States; used at 1:1000), and rabbit anti-ß-actin antibody (ABclonal, Wuhan, China; used at 1:50000) diluted in TBST (10% goat serum) at 4°C overnight. After incubation, the membranes were rinsed three times for 10 min in TBST. Then the membranes were incubated with HRP-conjugated goat anti-mouse IgG secondary antibody (ABclonal, Wuhan, China; used at 1:10000) or HRP-conjugated goat anti-rabbit IgG secondary antibody (ABclonal, Wuhan, China; used at 1:10000) in blocking solution for 1 h at room temperature, and washed further three times in TBS-T for 10 min. The protein bands were visualized using ECL Western-blot detection reagents (Millipore, United States), semi-quantified using Image J 1.51j8 software (National Institutes of Health, United States), and normalized to the corresponding band intensity of ß-actin.

### Immunofluorescence Staining

For immunofluorescence staining, tissue sections were washed with 0.1 M PBS for 10 min at room temperature. After rinsing with PBS, tissue sections were blocked with 10% normal goat serum in 0.1 M PBS containing 0.3% Triton X-100 for 2 h at room temperature and then incubated with the following primary antibodies overnight at 4°C, rabbit anti-SphK1 (Abcam, Cambridge, MA, United States; used at 1:500), mouse anti-SphK1 (Santa Cruz, United States; used at 1:500), mouse anti-S1PR2 (Santa Cruz Biotechnology, CA, United States; used at 1:500), rabbit anti-GFAP (Abcam, Cambridge, MA, United States; used at 1:1000), and rabbit anti-NeuN (Abcam, Cambridge, MA, United States; used at 1:1000). On the following day, tissue sections were washed three times for 10 min in 0.1 M PBS and then incubated for 2 h at room temperature with donkey Anti-Rabbit IgG H&L (Alexa Fluor^®^ 488, Abcam, United States; used at 1:500), and goat anti-mouse IgG H&L (Alexa Fluor^®^ 647, Abcam, United States; used at 1:500) conjugated secondary antibodies. Following this incubation period, tissue sections were washed three times with 0.1 M PBS for 10 min and then mounted with Vectashield DAPI Hardset mounting medium (Solarbio, China). Immunofluorescence staining pictures were captured employing a LSM800 confocal microscope (Zeiss, Germany).

### Immunohistochemistry

Frozen tissue sections were washed with 0.1M TBS for 10 min at room temperature. Antigen retrieval was achieved by microwaving the sections in 0.01M sodium citrate buffer for 10 min. Between all steps, the tissue sections were washed thoroughly using 0.1M TBS. Tissue sections were blocked with 5% BSA for 30 min at 37°C and followed by incubation with primary mouse anti-SphK1 antibody and mouse anti-S1PR2 overnight at 4°C. The next day, tissue sections were incubated with biotinylated-conjugated goat anti-mouse for 2 h at 37°C, followed by streptavidin-biotin complex (SABC) for 1 h at 37°C. Color reactions were performed using 5-bromo-4-chloro-3-indolylphosphate and nitroblue tetrazolium (BCIP/NBT). Tissue sections were air-dried and sealed with coverslips using the water-soluble mounting medium. SABC-AP kit was supplied by Boster Biological Technology (Wuhan, China). Images were acquired using an ordinary microscope (Carl Zeiss A1, Jena, Germany), and accompanying software for image acquisition.

### Quantification of Immunofluorescence Intensity and Image Analysis

Immunofluorescence quantification was performed using Image J software, following the Image J User Guide^1^ (NIH, Bethesda, MD). For calculating GFAP-positive cells, SphK1-positive cells, and S1PR2 -positive cells, five brain slices of each sample were analyzed in histological analysis using the Image J cell counting plugin tool (Chen et al., 2019; Tozaki-Saitoh et al., 2019).

### Statistical Analysis

All data from this study are presented as mean ± SD. The statistical analyses were performed using the statistical software SPSS, version 24.0 (Chicago, IL, United States), and GraphPad Prism software, version 8.2.1 (San Diego, CA, United States). One-way analysis of variance (ANOVA) with LSD *post hoc* test was used for multiple comparisons. Two-group analysis was performed using the Student’s *t* test. Clinical characteristics were compared between groups with the use of Student’s *t*-test or Fisher’s exact test. A value of *P* < 0.05 was accepted to indicate a statistically significant difference.

## Results

### Behavior and Seizures in the Pilocarpine Rat Model

**FIGURE 1:**
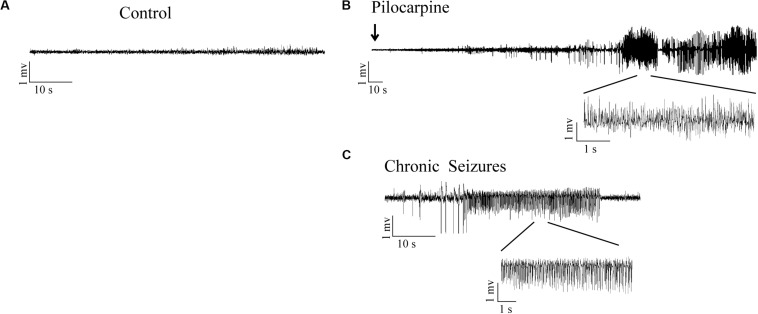
LFPs signals in hippocampal CA3 region in response to SE induction. **(A)** LFPs recordings in control rats. **(B)** Representative recordings of pilocarpine induced SE in epileptic rats. **(C)** Representative recordings of the pilocarpine induced chronic spontaneous seizures (day 30 after SE).

No obvious abnormal behavior was observed in rats after LiCl treatment. Within 10–15 min after the administration of pilocarpine, all rats developed a series of peripheral cholinergic symptoms, including pupil narrowing, diarrhea, mild tremors, salivation, weeping blood, and stereotype of movements such as paw licking, sniffing, and wet dog shakes at onset. About 20 min after pilocarpine injection, most rats experienced continues Racine’s stage IV-V seizures (Racine, 1972), characterized by rearing, falling, and generalized convulsions. After 60 min from SE onset, SE was quelled by the administration of diazepam (10 mg/kg, i.p.). Rats were individually housed in cages for further experiments. The pilocarpine rats experienced severe epileptic seizures during the first 72 h after pilocarpine administration (Figure 1). Rats were killed at different time points, corresponding to the period of epileptogenesis: the acute phase (6 h post-SE, 1 day post-SE, 3 days post-SE) (the rats experienced epileptic seizures), the latent phase (7 days post-SE) (the rats in this group did not display seizures between 4 days post-SE and 7 days post-SE), the chronic phase (30 days post-SE, 56 days post-SE) (the rats in this group presented recurring spontaneous seizures). No seizures were observed in rats from the control group.

### Clinical Characteristics

In this study, the examination of age, gender, and surgical resection site showed no significant difference between the subjects in the control and drug-resistant TLE groups. The control group included six males and four females with an average age of 27.0 ± 8.9 years. For the TLE group patients, the mean age was 26.6 ± 12.9 years, the mean duration of epilepsy was 11.2 ± 7.6 years, and contained nine males and seven females.

### Astrocyte Activation in Epileptic Rats and TLE Patients

**FIGURE 2:**
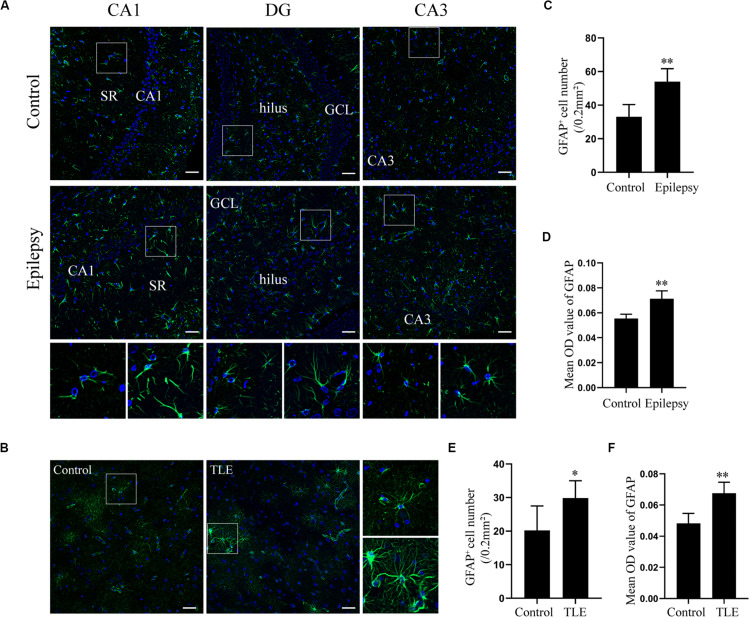
Astrocyte activation in the hippocampal section of pilocarpine-induced epileptic rats at day 7 after SE and the temporal cortex specimens of drug-resistant TLE patients. **(A)** Immunofluorescence staining showed the activation of astrocyte in the epileptic rat hippocampus. SR, stratum radiatum; GCL, dentate granule cell layer. Scale bar = 50 μm. **(B)** Immunofluorescence staining demonstrated the activation of astrocyte in the TLE patient temporal cortex. Scale bar = 50 μm. **(C)** GFAP positive cell number in rat hippocampus. **(D)** Quantitative analysis of the mean OD value of GFAP positive cells in rat hippocampus. **(E)** GFAP positive cell number in the patient temporal cortex. **(F)** Quantitative analysis of the mean OD value of GFAP-positive cells in the patient temporal cortex. Data are presented as mean ± SD (*n* = 4). Student’s *t*-test, ^∗^*P* < 0.05, ^∗∗^*P* < 0.01, versus the control group.

Glial fibrillary acidic protein (GFAP) is an intermediate filament predominantly expressed in astrocytes in the CNS (McCall et al., 1996). Astroglial activation is generally characterized by astrocyte hypertrophy, proliferation, and an increase in GFAP (Anderson et al., 2014). The expression of GFAP in epileptic rats and TLE patients was determined by immunofluorescence to elucidate the astrocyte reaction involved in epilepsy. Immunofluorescence analysis confirmed that astrocytes exhibit a strong increase of GFAP and hypertrophy in rat hippocampus 7 days post-SE and in the temporal cortex of TLE patients (Figures 2A,B). Quantitative analysis with Student’s *t*-test revealed that the mean optical density (OD) value of GFAP immunoreactivity in the rat hippocampus and in the TLE patient temporal cortex was significantly higher than that of the control group (Figures 2D,F) (^∗∗^*P* < 0.01). Further, there was a significant increase in the number of GFAP-positive cells in the rat hippocampus 7 days post-SE and the temporal cortex of TLE patients (Figures 2C,E) (^∗^*P* < 0.05, ^∗∗^*P* < 0.01).

### SphK1 and S1PR2 Expression in the Hippocampus After Pilocarpine Treatment

**FIGURE 3:**
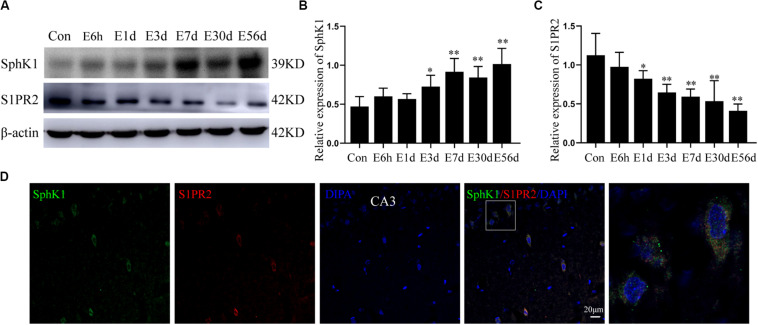
Hippocampal expression of SphK1 and S1PR2 after pilocarpine treatment. **(A)** Representative western blots identified SphK1 and S1PR2 in epileptic and control rats. **(B)** Quantitative analysis of the SphK1/ß-actin ratio (*n* = 4). **(C)** Quantitative analysis of the S1PR2/ß-actin ratio (*n* = 4). **(D)** Immunofluorescence staining for SphK1 (green) and S1PR2 (red) in hippocampal CA3 region 7 days after pilocarpine treatment. Data are presented as mean ± SD, one-way ANOVA with LSD *post hoc* test, ^∗^*P* < 0.05, ^∗∗^*P* < 0.01 versus the control group.

Consistent with our previous study, we first investigated whether epileptic seizures could affect the expression of SphK1 and S1PR2 protein levels (Figure 3A). We performed Western blot analysis to assess the temporal dynamics of SphK1 and S1PR2 protein levels in the hippocampus of control rats and rats subjected to SE (the acute phase, the latent phase, and the chronic phase), which were sacrificed at each time point (Figures 3A–C).

The level of SphK1 protein expression in epileptic rat hippocampus (3 days post-SE, latent phase, and chronic phase) was increased when compared with that in control rats (^∗^*P* < 0.05, ^∗∗^*P* < 0.01) (Figures 3A,B). In particular, the expression of SphK1 was increase at 3 days after pilocarpine treatment (Figure 3B). No differences with statistical significance were found in SphK1 levels in the acute phase group (6 h post-SE and 1 day post-SE) when compared with the control group (Figure 3B) (^∗^*P* > 0.05). This increase in SphK1 was similar to that previously seen at 48 h in the kainic acid (KA)-induced mice model (Lee et al., 2010).

In pilocarpine-induced epileptic rats, the S1PR2 protein level was decreased in the hippocampus (1 day post-SE, 3 days post-SE, latent phase, and chronic phase), and this downregulation persist for several weeks (Figure 3C) (*n* = 4, ^∗^*P* < 0.05, ^∗∗^*P* < 0.01). Statistical analysis showed no significant difference in the expression of S1PR2 protein between the control group and acute phase group (6 h post-SE) (*P* > 0.05).

In addition, immunofluorescence staining demonstrated that SphK1 (green) co-expressing with S1PR2 (red) in the hippocampus of epileptic rats. The two proteins predominantly co-localized in the cytoplasm, and small amounts were also present in the nucleus (Figure 3D).

### Cellular Localization of SphK1 in the Hippocampus From Epileptic Rats

**FIGURE 4:**
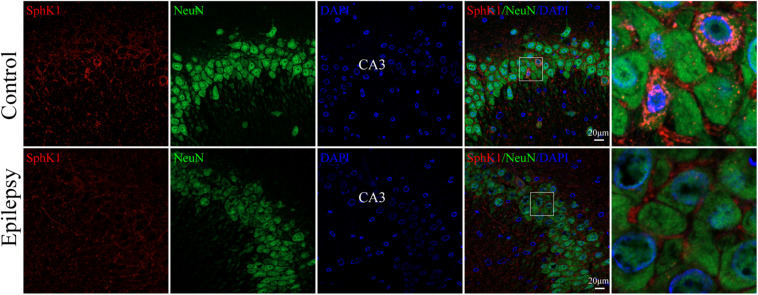
Cellular localization of SphK1 in CA3 region of the hippocampus in epileptic rats. **(left)** Confocal images showing co-localization of SphK1 and DAPI in neurons. **(right)** Enlarged view of indicated co-localization between SphK1 with neurons.

Our previous study showed that SphK1 signal was well co-expressed with the astrocyte marker GFAP in the hippocampus (Dong et al., 2019). On this basis, we further evaluate the SphK1 expression in the neuronal cells of hippocampal CA3 region. Immunofluorescence staining verified that SphK1 co-localized with neuronal dendrite-specific marker NeuN (green) in both control and epileptic rat hippocampus (Figure 4). Note that SphK1 was observed mainly on the neuronal membrane as well as in the cytoplasm of the control rats (Figure 4). As is well known, SphK1 is found to be present in the cytosol (Pitson et al., 2003). Accumulating evidence suggest that the activation of SphK1 is associated with its translocation to the plasma membrane (Pitson et al., 2005; Ceccom et al., 2014). In the epileptic rats (7 days post-SE), SphK1 was predominantly positive in the neuronal membrane and rarely in the cytoplasm (Figure 4). These results mentioned above lead us to the hypothesis that pilocarpine treatment might alter SphK1 translocation in neurons in CA3 fields of rat hippocampus. However, more studies are needed to confirm these findings.

### Cellular Localization of S1PR2 in the Hippocampus From Epileptic Rats

**FIGURE 5:**
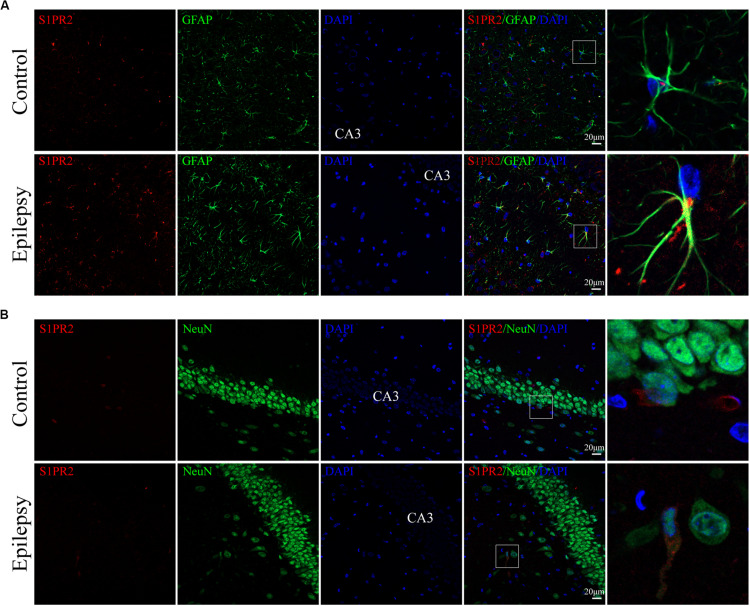
Cellular localization of S1PR2 in hippocampal tissues from epileptic rats. (**A**, left) Confocal images showing co-localization of S1PR2 and DAPI in astrocytes. (**A**, right) Enlarged view of indicated co-localization between S1PR2 with astrocytes. (**B**, left) Confocal images showing co-localization of S1PR2 and DAPI in neurons. (**B**, right) Enlarged view of indicated co-localization between S1PR2 with neurons.

We used immunofluorescence staining to evaluate the cellular localization of S1PR2 protein in the hippocampus of epileptic rats. It was noticeable that S1PR2 signal (red) co-located in both astrocyte cytoplasm and nucleus (green) in the control rat hippocampus (Figure 5A). We found that S1PR2 was expressed in the cytoplasm of astrocytes in rat hippocampal 7 days after pilocarpine injection (Figure 5A). In addition, we observed only a small amount of S1PR2 signal (red) co-localized with the neuronal cell marker NeuN (green) in both the control and epileptic rat hippocampal CA3 region (Figure 5B). Concomitantly, the number of hippocampal astrocytes was increased 7 days post-SE and showed hypertrophy of cell bodies as mentioned previously. These findings considered together indicate that SphK1 and S1PR2 were linked to a great degree with the activation of astrocyte in the epileptogenesis.

### Expression and Cellular Localization of SphK1 in Temporal Cortex From TLE Patients

**FIGURE 6:**
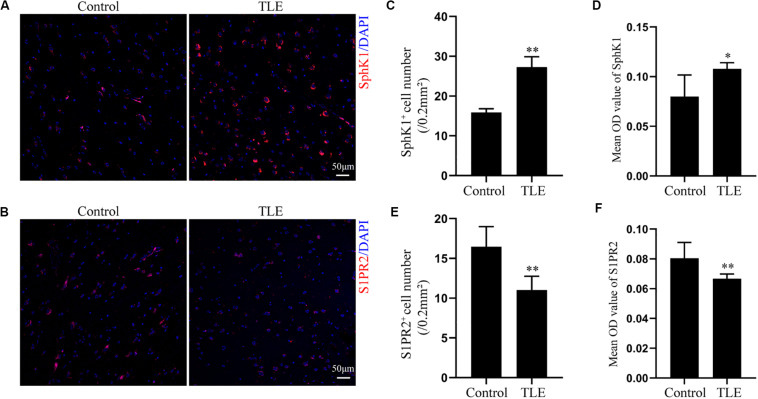
Expression of SphK1 and S1PR2 in the drug-resistant TLE patient temporal cortex. **(A)** Immunofluorescence staining demonstrated the SphK1 protein levels in the temporal cortex of TLE patients. **(B)** Immunofluorescence staining demonstrated the S1PR2 protein levels in the temporal cortex of TLE patients. **(C)** The number of SphK1-positive cells in the temporal cortex. **(D)** Quantitative analysis of the mean OD value of SphK1-positive cells in the non-epileptic patient temporal cortex and drug-resistant TLE patient temporal cortex. **(E)** The number of S1PR2-positive cells in the temporal cortex. **(F)** Quantitative analysis of the mean OD value of S1PR2-positive cells in the non-epileptic patient temporal cortex and drug-resistant TLE patient temporal cortex. Data are presented as mean ± SD, Student’s *t*-test, ^∗^*P* < 0.05, ^∗∗^*P* < 0.01 versus the control group.

**FIGURE 7:**
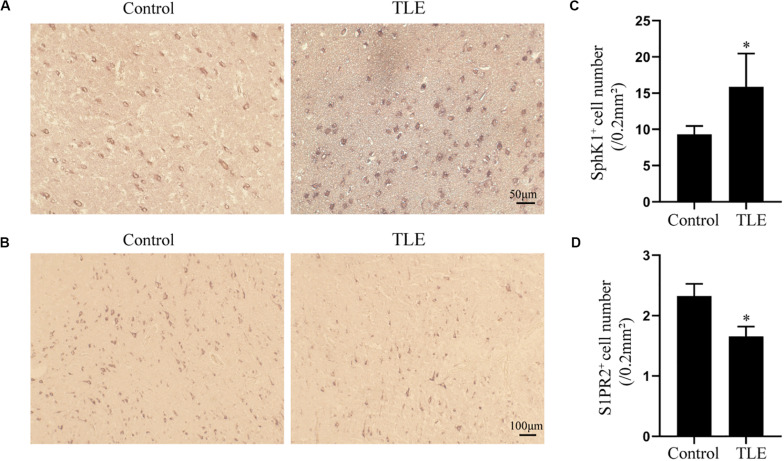
Immunohistochemical analysis of SphK1 and S1PR2 expression in drug-resistant TLE patient temporal cortex. **(A)** Representative SphK1 immunostaining in the temporal cortex specimens of control patients and drug-resistant TLE patients. **(B)** Representative S1PR2 immunostaining in the temporal cortex specimens of control patients and drug-resistant TLE patients. **(C)** Quantitative analysis of the number of SphK1-positive cells in the temporal cortex. **(D)** Quantitative analysis of the number of S1PR2-positive cells in the temporal cortex. Data are presented as mean ± SD, Student’s *t*-test, ^∗^*P* < 0.05 versus the control group.

**FIGURE 8:**
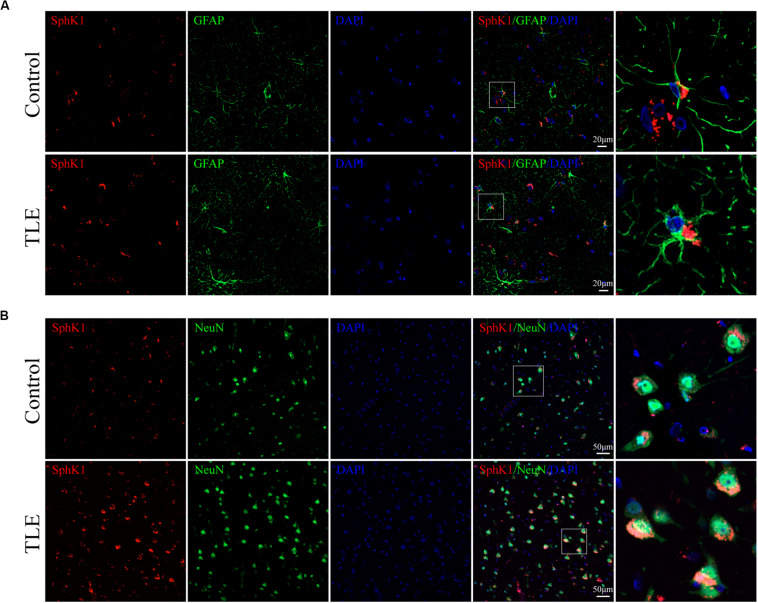
Cellular localization of SphK1 in temporal cortex tissues from patients with drug-resistant TLE patients. (**A**, left) Confocal images showing co-localization of SphK1 and DAPI in astrocytes. (**A**, right) Enlarged view of indicated co-localization between SphK1 with astrocytes. (**B**, left) Confocal images showing co-localization of SphK1 and DAPI in neurons. (**A**, right) Enlarged view of indicated co-localization between SphK1 with neurons.

Having characterized the spatial and temporal expression pattern of SphK1 protein in epileptic rats, we next determined SphK1 protein expression in the temporal cortex specimens obtained from patients with drug-resistant TLE patients through immunohistochemistry and immunofluorescence staining. Immunohistochemistry showed that SphK1-positive cells were expressed in the controls and TLE patient brain, and a higher level of SphK1 was detected in TLE specimens than in non-epileptic controls. The co-expression of DAPI with SphK1 positive cell number was significantly increased in the TLE specimens compared with that in the control specimens (Figures 6A,C) (^∗^*P* < 0.05, ^∗∗^*P* < 0.01), which was consistent with the immunohistochemistry result (Figures 7A,C) (^∗^*P* < 0.05). The optical density analysis revealed that the mean OD value of SphK1 in the brain temporal cortex was markedly increased in the TLE group compared with the control group (Figure 6D) (^∗^*P* < 0.05). Importantly, SphK1 was co-localized with GFAP, an astrocyte marker, and with NeuN, a neuronal marker in the non-epileptic controls and TLE patients (Figures 8A,B). Moreover, in both control and TLE patient temporal cortex, SphK1 primarily accumulates in the cytoplasm of the astrocyte and neuron.

### Expression and Cellular Localization of S1PR2 in Temporal Cortex From TLE Patients

**FIGURE 9:**
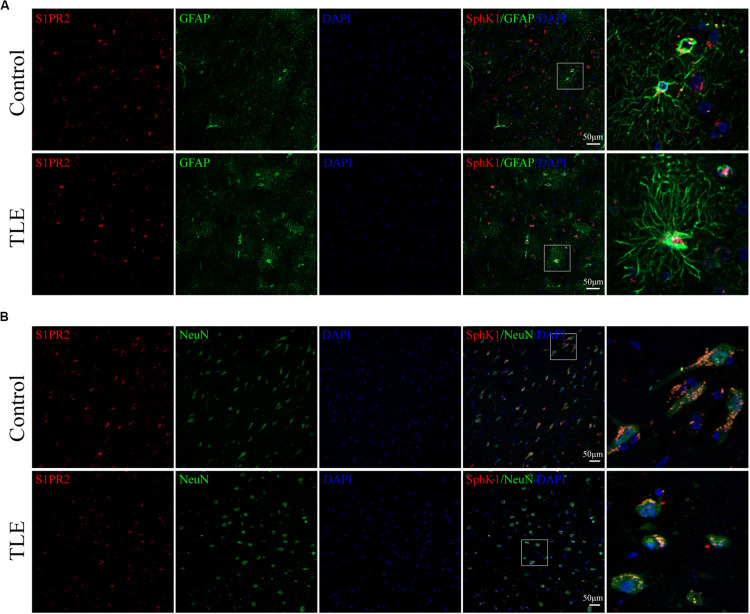
Cellular localization of S1PR2 in temporal cortex tissues from patients with drug-resistant TLE patients. (**A**, left) Confocal images showing co-localization of S1PR2 and DAPI in astrocytes. (**A**, right) Enlarged view of indicated co-localization between S1PR2 with astrocytes. (**B**, left) Confocal images showing co-localization of S1PR2 and DAPI in neurons. (**A**, right) Enlarged view of indicated co-localization between S1PR2 with neurons.

Cellular localization of S1PR2, evaluated by immunofluorescence, showed that in both controls and TLE patient temporal cortex specimens, S1PR2 signal (red) was observed in astrocytes (green) and neurons (green). Decreases in the amount of S1PR2 positive cells were observed on TLE patients in these temporal cortex fields when compared to the non-epileptic controls (Figures 6B,E, 7B,D) (^∗^*P* < 0.05, ^∗∗^*P* < 0.01). Immunofluorescence analysis demonstrated that the S1PR2 expression level was obviously reduced in TLE specimens compared with non-epileptic controls (Figure 6F) (^∗^*P* < 0.05). Our study also established that S1PR2 was widely expressed in the human temporal cortex and located in both astrocyte cytoplasm and nucleus, neuronal cytoplasm, and other brain cells (Figures 9A,B). In particular, S1PR2 was easily seen in the cell bodies of astrocytes whereas less discovered in nucleus in controls (Figure 9A). In TLE group, S1PR2 presented mainly in the astrocyte nucleus, except for a small amount in the cytoplasm (Figure 9A). The results also showed S1PR2 was not appreciably expressed in neuronal nucleus (Figure 9B).

## Discussion

In the present study, we have identified the spatiotemporal expression of SphK1 and S1PR2 protein in the pilocarpine rat model and the temporal cortex specimens from patients with drug-resistant TLE. To the best of our knowledge, we demonstrated for the first time that SphK1 was increased in epileptogenic foci of drug-resistant TLE patients and mainly located in astrocytes and neurons. In addition, the expression of S1PR2 was downregulated in specimens of drug-resistant TLE patients and widely expressed in astrocytes and neurons. Of note, the expression and localization of SphK1 and S1PR2 in the hippocampus of epileptic rats were similar to those observed in TLE patients’ temporal cortex specimens. These findings indicated that SphK1 and S1PR2 might be involved in the pathological mechanism of spontaneous seizures and might represent a potentially novel therapeutic target for seizure prevention.

Sphingosine kinase 1 and S1P receptors (S1PR1–S1PR5) have been widely acknowledged in the CNS. In particular, SphK1 is a rate-limiting enzyme that converts sphingosine into S1P through phosphorylation and plays a critical role in regulating cellular processes by affecting multiple downstream corresponding effector molecules. Most of the known actions of S1P are mediated by a family of five specific G protein-coupled receptors. However, little known about the role of SphK1 and S1PR2 in TLE.

Pilocarpine model in rats has been considered as a well-established model in TLE due to its capability to resemble the behavioral, electrographic, and the neuropathological features of human TLE (Turski et al., 1989). Rats injected with pilocarpine leads to SE and after a period time without seizures, finally develops into a chronic epileptic condition characterized as spontaneous recurrent seizures (Curia et al., 2008; Scorza et al., 2009; Ali et al., 2018). It is well known that astrocytes respond to neuronal activity and has been proposed as an accurate detector of neuronal activity (Araque et al., 2002; Guo et al., 2007). In the control group, the astrocyte processes were long and radially ranged. We observed astrocyte activation, as assessed by overexpression in GFAP immunoreactivity and demonstrated larger cell body and long processes, throughout the epileptic rat hippocampus and the TLE patient temporal cortex. Reactive astrocytes may exhibit beneficial effects on neuronal survival by several biological processes in the early stage of CNS injury. However, the prolonged and increased activation of astrocyte might contribute to epilepsy and be closely related to epilepsy induced neuronal death (Meng et al., 2016).

Recent studies have verified that SphK1 is implicated in the process of inflammation and causes chronic neuroinflammation (Melendez, 2008; Dai et al., 2018). Importantly, a recent study has reported that upregulates the SphK1/S1P receptor signaling pathway may be a key factor of astrocytes mediated chronic inflammation in multiple sclerosis, and BBR ameliorated the severity of MS symptom in mouse model through inducing the increase in SphK1 and S1P. We detected high levels of SphK1 expression in the brain specimens of drug-resistant TLE patients and epileptic rats. In epileptic rats, SphK1 significantly increased 3 days post-SE in the hippocampus, which was consistent with a previous study showing that the stronger immunoreactivity in mice hippocampal 48 h after KA treatment (Lee et al., 2010). It is suggested that SphK1 may play a role in pilocarpine-induced initial brain injury. In addition, SphK1 exhibited higher expression levels in the latent phase and chronic phase in the epileptic rat hippocampus compared with that in the controls. These data indicate that the dysregulation of SphK1 may promote the formation of epileptic foci associated with the pathogenic mechanism of spontaneous seizures. A previous report showed that SphK1 and S1PR3 expression were increased in LPS-activated astrocytes and suggested that the SphK1/S1PR3 signaling axis might mediate and amplify the inflammatory in various CNS disorders (Fischer et al., 2011). A recent study has also reported that genetic deletion of SphK1 results in the milder disease course, which associate with the suppression of glial cell proliferation and astrogliosis in the Sandhoff model mouse (Wu et al., 2008). We identified that SphK1 was expressed by reactive astrocytes and neurons in the epileptic rat hippocampus, as demonstrated by partial co-localization of GFAP in cytoplasm and co-labeling with NeuN in neuronal plasma membranes and cytoplasm. It is generally acknowledged that SphK1 must translocate from the cytoplasm to the plasma membrane to mediate pro-proliferative and pro-survival signal through the generation of S1P. Immunofluorescence showed that SphK1 was expressed much lighter in control rat neuronal cell cytoplasm. While this change was not statistically significant, it suggests changes in the SphK1 subcellular localization after pilocarpine treatment. The mechanism by which SphK1 regulates seizures is uncertain, but our data suggest the possibility that the effect occurs via astrocyte activation and hypertrophy. We confirmed that SphK1 expression was increased in TLE patient temporal cortex. Both astrocytes and neurons can express SphK1 and release S1P (Anelli et al., 2005). A possibility is that astrocytes and neurons, as a result of brain insult, may release S1P locally to induce astrocyte activation via S1PRs in autocrine or paracrine manner at the region of injury (Mullershausen et al., 2007; Wu et al., 2008).

Preferential expression of S1PR2 (also referred to as H216, EDG-5, AGR16, and LP_B2_) in differentiating embryonic neurons and their extending axons was previously describes in rat embryo cortex tissue (MacLennan et al., 1997). In addition, it has recently been reported that the deletion of the S1PR2 gene in mice leads to spontaneous seizures accompanied by ictal-like EEG abnormalities and hyperexcitable pyramidal neurons, implying a vital role for S1PR2 in neuronal excitability (MacLennan et al., 2001). Furthermore, one report has shown that S1PR2 deficiency may cause severe seizures in juvenile mice and lead to CNS indults in surviving adults, such as enhanced gliosis in the hippocampus and cortex and impair some specific CNS function (Akahoshi et al., 2011). All aforementioned results support that the blockade of the S1PR2 signaling pathway can contribute to seizures; however, the spatiotemporal expression has not been illuminated in current epilepsy models and clinical patients. The present study describes for the first time a decreased S1PR2 expression in pilocarpine induced rat hippocampus and show that the similar phenomenon in drug-resistant TLE patient temporal cortex by immunohistochemistry and immunofluorescence. Moreover, the level of S1PR2 was declined 1 day post-SE as well as in the latent phase and chronic phase. We have further corroborated the co-localization of S1PR2 in activate astrocytes and neurons in both rat hippocampus and human temporal cortex. Therefore, depending on the brain region, species, and epilepsy phase, subcellular localization of S1PR2 might be different, a hypothesis that remains to be proved in the future. Taken together, these results hind that S1PR2 signaling axis regulates astrogliosis and proliferation and neuronal excitability in TLE pathogenesis.

In summary, our study demonstrates altered spatiotemporal expression of SphK1 and S1PR2 in the epileptic rat hippocampus and TLE patient temporal cortex. In the future, we will perform experiments in different animal species and animal seizure models, and further to investigate the effect of SphK1/S1PR2 signal specific inhibitor and agonists on molecules associated with epilepsy. These findings will provide new discovery of the SphK1/S1PR2 signaling pathway, identify important mechanisms contribute to TLE, and suggest a novel therapeutic target to promote remission for TLE and other CNS diseases mediate by SphK1/S1PR2 signaling pathway.

## Data Availability Statement

All datasets generated for this study are included in the article.

## Ethics Statement

Animal procedures were approved by the Jining Medical University Animal Ethics Commission. This study was compliant with the Declaration of Helsinki and approved by the Ethics Committee of Affiliated Hospital of Jining Medical University. Written informed consent was obtained from each patient or their lineal relatives.

## Author Contributions

Y-YD performed the experiments, analyzed the data, and wrote the manuscript with the association of MX and LW. S-SM and Q-BL recruited the participants and collected the specimens. SC and J-CZ prepared the figures. Q-XK and Y-KZ conceived and associated the research. All of the authors read and approved the final manuscript.

## Conflict of Interest

The authors declare that the research was conducted in the absence of any commercial or financial relationships that could be construed as a potential conflict of interest.

## Footnotes

1 https://imagej.nih.gov/ij/
